# Capillary blood reference intervals for platelet parameters in healthy full-term neonates in China

**DOI:** 10.1186/s12887-020-02373-6

**Published:** 2020-10-10

**Authors:** Dongyan Cui, Yan Hou, Ling Feng, Guo Li, Chi Zhang, Yanli Huang, Jiubo Fan, Qun Hu

**Affiliations:** 1 Department of Paediatric Haematology and Oncology, Tongji Hospital, Tongji Medical College, Huazhong University of Science and Technology, Wuhan, 430030 Hubei Province People’s Republic of China; 2 Department of Paediatrics, Xiangyang Central Hospital, Xiangyang, 441021 Hubei Province People’s Republic of China; 3 Department of Gynaecology and Obstetrics, Tongji Hospital, Tongji Medical College, Huazhong University of Science and Technology, Wuhan, 430030 Hubei Province People’s Republic of China; 4 Department of Clinical Laboratory, Tongji Hospital, Tongji Medical College, Huazhong University of Science and Technology, Wuhan, 430030 Hubei Province People’s Republic of China; 5 Department of Gynaecology and Obstetrics, Xiangyang Central Hospital, Xiangyang, 441021 Hubei Province People’s Republic of China; 6 Department of Clinical Laboratory, Xiangyang Central Hospital, Xiangyang, 441021 Hubei Province People’s Republic of China

**Keywords:** Neonates, Capillary blood, Platelet count, Platelet parameters, Reference intervals

## Abstract

**Background:**

No consensus has been reached on capillary blood reference intervals for platelet parameters in full-term neonates. We aimed to establish neonatal capillary blood reference intervals for platelet parameters and evaluate influences of sex, gestational age and postnatal age on platelet parameters.

**Methods:**

This study was a prospective investigation and implemented in 594 healthy full-term neonates from 12 to 84 h of age, using SYSMEX XN-9000 haematology automatic analyser by means of capillary blood. Reference intervals for platelet parameters were defined by an interval of 2.5th − 97.5th percentiles.

**Results:**

Capillary reference interval for platelet count was (152–464) × 10^9^/L. No significance was found between sex-divided reference intervals for platelet parameters. The values of platelet count changed minimally across gestational age (37–41 weeks) and postnatal age (12–84 h). Reference intervals for other platelet parameters were affected by these factors to a different extent.

**Conclusions:**

We established capillary blood reference intervals for platelet parameters in the first days after birth of full-term neonates in China.

## Background

Reference intervals (RIs) play a critical role in clinical practice. Appropriate RIs for platelet parameters ensure clinical laboratories provide reliable information and enable clinicians to correctly interpret results and further determine whether transfusions are needed for neonates [1, 2]. However, few normative data are available in RIs for platelet parameters in full-term neonates [3]

Neonates are in a crucial period of rapid development, and platelet parameters are significantly affected by these physiological changes [4]. Moreover, RIs for platelet parameters have often been derived from haematological results of both inpatient and outpatient neonates [4, 5], or blood samples are leftover material from the donated blood for a specific use in a pre-term (delivered at less than 37 weeks of gestation) or full-term neonate population [6, 7], which may be inaccurate and unreliable for full-term neonates. It is hard for neonatal health care providers to provide standard-of-care health services as a lack of appropriate RIs for haematology may impede diagnostics for neonates [8]. The study of RIs for platelet parameters in neonates is highly restricted due to the ethical limitations and difficulties to obtain sufficient blood samples. Capillary sampling by the automated incision device spends a shorter time for blood collection with reduced haemolysis [9], and is increasingly used in clinical practice nowadays in China. Capillary blood for haematology tests is as small as necessary in volumes and as non-invasively as possible with a very low frequency of local infection and decreased extent of bruising [10]. Although capillary blood is used to develop RIs for other biologic markers [11–13], studies have not been carried out to evaluate its clinical utility in neonatal capillary blood RIs for platelet parameters.

Ideally, RIs for platelet parameters are established in a healthy full-term neonate population and more than 120 specimens are required by the Clinical and Laboratory Standards Institute (CLSI) [14]. The objectives of the present study were to determine capillary blood RIs for platelet count (PLT) and related parameters in 594 healthy full-term neonates from 12 to 84 h of age, and furthermore to evaluate influences of sex, gestational age and postnatal age on platelet parameters.

## Methods

### Study populations

The study commenced after obtaining Institutional Ethical Committee approval (TJ-IRB20190308). The subjects were prospectively enrolled into this study, and all of them were healthy full-term (259 to 293 days (37 to 41 weeks) of gestation) neonates born at two hospitals from November 2018 to April 2019. Major inclusion/exclusion criteria are listed as follows. As listed in Chang et al. [6] for mothers, maternal age ranged from 20 to 40 years old and they had a normal health check-up without unfavourable past history during pregnancy, such as smoking, infectious diseases (hepatitis B/C, HIV, syphilis infections), idiopathic thrombocytopenic purpura, malignancies and chronic diseases (diabetes mellitus, autoimmune disease, immunodeficiencies and thrombotic disorders); mothers did not receive aspirin during pregnancy; a vaginal delivery or caesarean section was event-free. Professional obstetricians were responsible for the data of this part. As listed in Chang et al. [6] and Wasiluk [15] for neonates, they fitted all clinical state criteria for full-term neonates; they were born with normal birth weight (2500-4000 g) appropriate for gestational age and normal Apgar score (8–10 points at 1–5 min of life); physical examinations of the subjects were normal without any signs of infection or congenital anomalies at the time of sampling. And we excluded all neonates with an unfavourable perinatal history of premature rupture of membrane more than 24 h, abnormal placenta, placenta abruption, chorioamnionitis or meconium stain, or with a medical history of hospitalizing in the neonatal intensive care unit. All subjects are Han ethnicity.

### Blood sampling

After written informed consent was obtained from a parent or guardian for all subjects, we collected about 70 μL capillary whole blood of the subjects during the first 4 days of life (12 to 84 h old), by heel prick with the automated incision device into BD Microtainer tubes (spec, 0.5 mL; Becton Dickinson and Company, USA) containing K2-ethylenediaminetetraacetic acid (EDTA) as an anticoagulant. Samples were obtained by experienced technicians at the same time of blood sampling for the screening of congenital hypothyroidism and phenylketonuria or the routine serum bilirubin in morning hours (1–2 h later after last food intake). The first-second drop of blood was discarded and each collection time was less than 1 min. Samples were stored at ambient temperature for a maximum of 4 h before the assay was performed.

### Laboratory analyses

All capillary blood samples were analysed by SYSMEX XN-9000 haematology automatic analyser (Sysmex Corporation, Kobe, Japan) in the pre-dilution mode (dilution ratio, complete blood: PK dilution = 1:6) to measure PLT (× 10^9^/L) and related parameters, including mean platelet volume (MPV, fL), plateletcrit level (PCT, %), platelet size distribution width (PDW, fL) and platelet large cell ratio (P-LCR, %). As listed in Kaito et al. [16] for the detection principle of platelet parameters, MPV was calculated by a formula (PCT / PLT × 10^4^), and PDW and P-LCR were analysed from a histogram of platelet size distribution. Quality controls were run during every shift.

### Statistical analysis

Data management and analyses were performed using SPSS 25.0 software (SPSS Inc., Chicago, USA). The data distribution was evaluated using Q-Q plots, histograms and Shapiro-Wilk test. We built Box plot (with Tukey variation) to identify the outliers using GraphPad Prism 8.2.1 (GraphPad software, La Jolla, CA, USA). An outlier was defined as a value < Q_1_–1.5 × IQR (Q_1_: 25th percentiles, IQR: interquartile range), or > Q_3_ + 1.5 × IQR (Q_3_: 75th percentiles). Quantitative data were expressed as either mean (± standard deviation, SD) for normally distributed data or median (IQR) for data not normally distributed. RIs for platelet parameters were defined by an interval of 2.5th – 97.5th percentiles. We performed Student’s *t*-test or Mann-Whitney U test as appropriate in order to evaluate influence of sex on platelet parameters. Pearson’s or Spearman’s correlation coefficients was performed as appropriate to evaluate correlations between platelet parameters and correlation factors. Two-tailed *P* values of less than 0.05 were set as statistical significance.

## Results

### Platelet parameters reference intervals

**Fig. 1:**
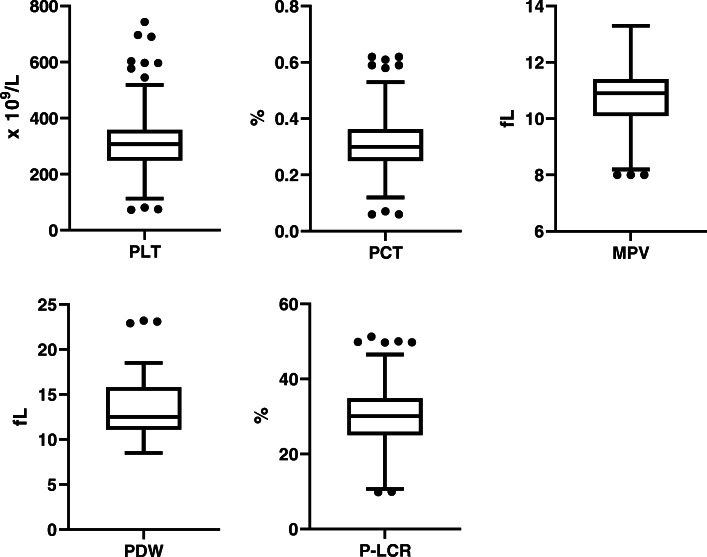
The Box plot for platelet parameters data. The centre, the variability and the outliers are displayed. The following number of outliers was found: 11 for PLT, 9 for PCT, 3 for MPV, 3 for PDW, and 7 for P-LCR. Abbreviations: PLT, platelet count; PCT, plateletcrit; MPV, mean platelet volume; PDW, platelet size distribution width; P-LCR, platelet-large cell ratio

Totally 594 neonates were enrolled in present study. Demographic data are shown in Table 1. Several values were identified as outliers in platelet parameters and we excluded cases test-by-test in data analyses (see Fig. 1). Except for PLT, other platelet indices were not normally distributed. RIs for platelet parameters represent the central 95 % out of all data (Table 2). As a result, capillary blood RI of PLT ranged from 152 to 464 (× 10^9^/L).

**Table 1:** Demographic data of the subjects

Sex	Total	Male	Female
N	594	323	271
Gestational age, days	273 (266–278)	271 (265–278)	274 (268–279)
Postnatal age, hours	29 (22–49)	29 (22–49)	30 (22–49)

Quantitative data were expressed as median (*IQR* Interquartile range)

**Table 2:** Capillary blood reference intervals for platelet parameters in healthy term neonates

Platelet parameters	Percentiles		Demographic data		
	2.5th	97.5th	Total	Male	Female
PLT, ×10^9^/L	152	464	304 (± 77)	299 (± 77)	310 (± 77)
PCT, %	0.18	0.46	0.30 (0.26–0.36)	0.30 (0.25–0.36)	0.31 (0.26–0.37)
MPV, fL	8.3	12.6	10.9 (10.1–11.4)	10.9 (10.2–11.4)	10.8 (10.0–11.4)
PDW, fL	9.4	17.1	12.5 (11.1–15.8)	12.5 (11.2–15.8)	12.3 (11.0–15.9)
P-LCR, %	15.4	42.8	30.0 (25.0–34.7)	30.5 (25.2–35.2)	29.5 (24.7–34.0)

Abbreviations: *PLT* Platelet count; *PCT* Plateletcrit; *MPV* Mean platelet volume; *PDW* Platelet size distribution width; *P-LCR* Platelet-large cell ratio. Data were expressed as either mean (*SD* ± Standard deviation) or median (*IQR* Interquartile range)

### Correlation factors

Female neonates had higher capillary blood PLT values (310 (±77) × 10^9^/L) than male neonates (299 (±77) × 10^9^/L) but this difference was not statistically significant (*p-value* = 0.082). Expectedly, other platelet parameters were not significantly affected by sex (all *p-values* > 0.05) (Table 2). In order to evaluate correlations between platelet parameters and gestational age (259 to 293 days of gestation) and postnatal age (12 to 81 h old), we performed Pearson’s correlation coefficient in PLT and Spearman’s correlation coefficient in platelet indices (Table 3). Significant negative correlations between gestational age and platelet parameters were observed (*p-value* < 0.05). However, in view of the low coefficients of correlation in PLT and PDW across gestational age, a biologic relationship between them is likely absent. Moreover, correlations between postnatal age and platelet parameters (except for PCT) were significant but very mild as all of absolute values of correlation coefficients were below 0.2.

**Table 3:** Correlations between platelet parameters and gestational age and postnatal age in healthy term neonates

Factor		PLT	PCT	MPV	PDW	P-LCR
Gestational age	r^a^	−0.143	− 0.237	− 0.255	− 0.161	−0.206
	p	0.001	< 0.001	< 0.001	< 0.001	< 0.001
Postnatal age	r^a^	−0.085	0.027	0.094	0.196	0.105
	p	0.041	0.509	0.023	< 0.001	0.011

Abbreviations: *PLT* Platelet count; *PCT* Plateletcrit; *MPV* Mean platelet volume; *PDW* Platelet size distribution width; *P-LCR* Platelet-large cell ratio. ^a^. Pearson’s correlation coefficient was performed in PLT, and Spearman’s correlation coefficient was performed in other platelet indices

## Discussion

RIs for platelet parameters are of great value in diagnosis and monitoring of various diseases in the neonatal period, particularly in the first days of life [3, 17, 18]. Previous studies on developing RIs for platelet parameters provided important information [8, 19–25]. However, the RIs were based on cord blood or venous blood in pre-term or term neonates [4–6, 26]. In present study, we presented the first RIs for platelet parameters using capillary blood in healthy term neonates.

Unfortunately, due to the difficulty to persuade parents to obtain venous blood samples from their healthy babies, we did not determine difference among various sampling sites. Early studies [19, 27] reported that significantly lower PLT values were found in capillary blood than venous blood. Their data proved the fact that capillary MPV values were higher than venous MPV values. Not surprisingly, PCT values were positively correlated strongly with PLT values, while other platelet indices remained unchanged basically and did not obviously fluctuate along with PLT values [21]. Thus, a higher PCT value was also observed in venous blood compared with capillary blood. These data confirmed the effect of different sampling sites on platelet parameters in the neonatal period. Venous blood collection in neonates is full of challenges and time-consuming, which may make platelets relatively more likeness to activate and aggregate. Composition of capillary blood may be affected by local metabolic state as well as perfusion. Moreover, various extent of stress about extrusion on heel sampling sites may disturb the microcirculation and microenvironment and also affect the composition of capillary blood for that the samples may be mixed with an undetermined proportion of interstitial and intracellular fluids [26]. And in clinical practice, we should take neonatal sampling sites into consideration when choosing RIs for platelet parameters.

A recent study showed that neonatal age had a significant effect on the parameters regarding coagulation [28]. And we found a slight variation in PLT in the first days of life. Of note, a multicentre study recruiting a large neonate cohort [4] reported that PLT values changes with two peak sinusoids at 2–3 weeks and 6–7 weeks of postnatal age. On the other hand, in our current study, a very mild negative correlation was observed between PLT values and term gestational age (Pearson’s correlation coefficient, *r* = − 0.143), which may reflect a true absence of a biologic relationship. The multicentre study above and other previous studies [25, 29] reported that PLT values increased linearly with advancing gestational age. However, these studies [4, 30] are heterogeneous in nature because of both pre-term and term babies included and using variable blood samples, which may potentially lead to multiple confounding factors influencing their results and further resulting in a different observation with us.

The present study has some limitations. First, our cohort did not precisely determine the difference between various sampling sites because of the difficulty to obtain venous blood samples from healthy babies as described above. Secondly, as the neonates in our reference population aged from 12 to 84 h, we cannot extrapolate how the RIs vary in the first 12 h of life or after postnatal age of 84 h. Moreover, when the population is divided according to gestational age and postnatal age, the size of our study population does not meet the CLSI recommendation and therefore, we chose to report RIs for the combined population.

## Conclusions

In conclusion, for the first time, the current study established capillary blood RIs for platelet parameters in the first days after birth in China. Furthermore, sex difference was of little clinical significance and therefore RIs for platelet parameters were combined for male and female neonates. And our findings showed values of platelet parameters changed with advancing gestational and postnatal age, which might provide clues for further multicentre investigations aimed at offering more accurate RIs for haematological parameters, in a larger population study.

## Data Availability

All data generated or analysed during this study are included in this published article. The datasets used and/or analysed during the current study are available from the corresponding author on reasonable request.

## Abbreviations

RIs: Reference intervals
CLSI: Clinical and laboratory standards institute
EDTA: K2-ethylenediaminetetraacetic acid
PLT: Platelet count
MPV: Mean platelet volume
PCT: Plateletcrit level
PDW: Platelet size distribution width
P-LCR: Platelet large cell ratio
SPSS: Statistical product and service solutions
SD: Standard deviation
IQR: Interquartile range
